# Former smoking associated with epigenetic modifications in human granulosa cells among women undergoing assisted reproduction

**DOI:** 10.1038/s41598-024-54957-2

**Published:** 2024-02-29

**Authors:** Ziyin Tang, Audrey J. Gaskins, Robert B. Hood, Jennifer B. Ford, Russ Hauser, Alicia K. Smith, Todd M. Everson

**Affiliations:** 1 Gangarosa Department of Environmental Health, Rollins School of Public Health, Emory University, Atlanta, GA USA; 2 Department of Epidemiology, Rollins School of Public Health, Emory University, Atlanta, GA USA; 3 Department of Environmental Health, Harvard T.H. Chan School of Public Health, Harvard University, Boston, MA USA; 4 Department of Obstetrics and Gynecology, School of Medicine, Emory University, Atlanta, GA USA

**Keywords:** Former smoking, Epigenetics, DNA methylation, Granulosa cells, Epigenetics, Environmental impact

## Abstract

Smoking exposure during adulthood can disrupt oocyte development in women, contributing to infertility and possibly adverse birth outcomes. Some of these effects may be reflected in epigenome profiles in granulosa cells (GCs) in human follicular fluid. We compared the epigenetic modifications throughout the genome in GCs from women who were former (N = 15) versus never smokers (N = 44) undergoing assisted reproductive technologies (ART). This study included 59 women undergoing ART. Smoking history including time since quitting was determined by questionnaire. GCs were collected during oocyte retrieval and DNA methylation (DNAm) levels were profiled using the Infinium MethylationEPIC BeadChip. We performed an epigenome-wide association study with robust linear models, regressing DNAm level at individual loci on smoking status, adjusting for age, ovarian stimulation protocol, and three surrogate variables. We performed differentially methylated regions (DMRs) analysis and over-representation analysis of the identified CpGs and corresponding gene set. 81 CpGs were differentially methylated among former smokers compared to never smokers (FDR < 0.05). We identified 2 significant DMRs (*KCNQ1* and *RHBDD2*). The former smoking-associated genes were enriched in oxytocin signaling, adrenergic signaling in cardiomyocytes, platelet activation, axon guidance, and chemokine signaling pathway. These epigenetic variations have been associated with inflammatory responses, reproductive outcomes, cancer development, neurodevelopmental disorder, and cardiometabolic health. Secondarily, we examined the relationships between time since quitting and DNAm at significant CpGs. We observed three CpGs in negative associations with the length of quitting smoking (*p* < 0.05), which were cg04254052 (*KCNIP1*), cg22875371 (*OGDHL*), and cg27289628 (*LOC148145*), while one in positive association, which was cg13487862 (*PLXNB1*). As a pilot study, we demonstrated epigenetic modifications associated with former smoking in GCs. The study is informative to potential biological pathways underlying the documented association between smoking and female infertility and biomarker discovery for smoking-associated reproductive outcomes.

## Introduction

Cigarette smoking has been well associated with female infertility^1,2^. Both women who are current smokers and those who have quit smoking before conception were found to have lower natural fertility^3^. Previous studies have well-associated smoking with ovarian dysfunction as reflected clinically by lower ovarian reserve, poorer response to ovarian stimulation, and earlier age at menopause^4^. However, the underlying biological mechanisms remained to be further investigated.

Epigenetics is a useful tool for understanding the biological responses of environmental exposure to adverse health impacts and providing insights into biomarker development. Mounting studies associated smoking with changes in DNA methylome in a variety of biosamples, including the placenta, fetal cord blood, peripheral blood, and lung tissue, some of which were found to be persistent into childhood and adolescence^5–11^. To our best knowledge, none has investigated the smoking-associated epigenetic modifications in human granulosa cells (GCs) obtained from follicular fluid (FF).

Human FF is responsible for the constant and sufficient supply of nutrients to the oocyte, contributing to its maturation and good quality, which is a key element for quality of embryo and subsequent success of in vitro fertilization (IVF) treatment^12,13^. IVF is the most common assisted reproductive treatment (ART) procedure performed. The sources of FF mainly include the secretory activity of GCs and thecal cells, and blood plasma composition that crosses the thecal capillaries^14^. A recent study within women undergoing IVF reported that cigarette smoking was associated with de-regulation of 26 oxidative stress-related genes in cumulus cells originating from relatively undifferentiated GCs^15^. They also observed significantly lower methylation at promoter of interleukin-6 (IL-6) which is an important inflammation factor. It is plausible that DNAm altered by smoking exposure in these cells may affect the FF microenvironment, and as a consequence having influences on oocyte dysfunction. Therefore, interrogation on the association of smoking with DNAm in GCs may be informative about biological mechanisms underlying smoking-associated female infertility demonstrated by oocyte quality and dysfunction. In addition, the aspiration of oocytes from follicle is accompanied by FF during IVF treatment. Thus, GCs in FF is an ideal and reasonable biosample for screening of oocyte quality and dysfunction, particularly among those undergoing IVF. There are growing number of studies focused on discovery of protein and microRNA biomarkers for IVF in FF but less on DNAm biomarkers^13,16,17^.

We performed a pilot study consisting of 59 women undergoing ART who were non-active smokers at recruitment. This provides a unique opportunity to study the impacts of smoking, after cessation, on DNAm in GCs. We aimed to investigate the associations of former smokers versus never smokers with DNAm differences, which may have implications for better understanding the biological pathways through which prior smoking leads to ovarian dysfunction and providing insights into biomarker discovery for smoking-associated reproductive outcomes.

## Methods

### Study population

The study population in our analysis was a subset of participants in the Environment and Reproductive Health (EARTH) Study, a prospective cohort that was established for evaluating factors that influence human fertility and pregnancy outcomes^18^. Specifically, the participants consisted of women between 18 and 45 years of age who sought fertility evaluation and treatment at the Massachusetts General Hospital (MGH) Fertility Center (2004–2019). Infertility insurance coverage in Massachusetts requires smoking cessation prior to treatment. Therefore, current smokers could enroll in the EARTH Study (prior to starting infertility treatment) but were excluded in this analysis since it was unclear how recently they had quit smoking between joining EARTH and prior to starting IVF. At enrollment, participants completed questionnaires for demographics, lifestyle, and medical and reproductive history. Anthropometric measurements were collected by trained research staff and used to determine body mass index (BMI). For this pilot study, eligible participants were women who underwent an ART cycle between 2006 and 2016 and provided a FF sample during oocyte retrieval process. The details of participant inclusion criteria for this study are provided elsewhere^19^.

The institutional review boards of the Massachusetts General Hospital and Harvard T.H. Chan School of Public Health approved this study and all subjects met with trained study staff before providing written informed consent. All methods in the study were conducted in adherence to relevant guidelines and regulations.

### Follicular fluid collection

Oocyte retrieval occurred after 9–14 days of controlled ovarian stimulation. FF was retrieved from the first three follicles using a 16 G needle, with separate tubes for each sample containing 1 ml of flushing media. FF was then centrifuged to separate the supernatant and cellular pellet, and stored at − 80 °C. The stored pellet from the first aspirated follicle was shipped to the Emory Integrated Genomics Core on dry ice.

### Quality control and processing of DNA methylation data

DNA was extracted using the QIAamp UCP DNA Micro Kit (Qiagen, Hilden, Germany), and quantification was performed with Quant-iT dsDNA broad range assay kit (ThermoFisher, Waltham, MA), and assessed for quality on a 2% agarose gel. DNA preparation followed the Illumina Infinium HD Assay Methylation Protocol Guide. To limit potential batch effects, samples were randomly distributed across the plate. The Emory Integrated Genomics Core performed bisulfite modification with the EZ DNA Methylation Kit (Zymo Research, Irvine, CA), DNAm was quantified across the genome with the Illumina MethylationEPIC Beadarray (Illumina, San Diego, CA) following the manufacturer’s protocol. We utilized ‘*minfi’* package for quality control and pre-processing, and applied their method for detection *p*-values^20^. To reduce technical artefacts and probe type bias, we performed functional normalization and beta-mixture quantile (BMIQ) normalization^21,22^. We excluded probes on the X and Y chromosomes and those exhibiting low variability (with a standard deviation of beta-values < 0.02)^23^.

### Accounting for unmeasured confounding

We also aimed to control for technical confounders and cellular composition in our data, but given the small sample size we only had one batch and there is no reference methylome for GCs at this time. Thus, we utilized surrogate variable analysis (*sva* package in R) to estimate major sources of variation in our data, some of which are likely reflective of differences in cellular composition between samples, technical artefacts, or other unmeasured confounders. The first three surrogate variables were included in our models.

### Statistical analyses

#### Epigenome-wide association study (EWAS): identification of former smoking-associated CpGs

We performed an epigenome-wide association study (EWAS) using robust linear regressions for each CpG sites, with the methylation beta-values (continuous) as dependent variables and the smoking history (2-level factor: former vs. never smokers) as the independent variable, while adjusting for age, ovarian stimulation protocol (3-level factor: antagonist, flare, and luteal phase agonist) and three surrogate variables. A Manhattan plot was generated to summarize the EWAS results. Multiple tests were corrected by Benjamini-Horchberg (BH) procedure. Associations within false discovery rate (FDR) of 5% were considered statistically significant.

To test the robustness of the EWAS results, we performed sensitivity analyses that included additional adjustments for education levels (3-level factor: did not graduate from college, college degree, and graduate degree) and BMI (continuous, kg/m^2^) and compared the effect coefficients before and after the further adjustments. We limited this sensitivity analysis to the CpG sites that met the FDR of 5% from the main analysis. All analyses were performed in R version 4.1.0.

#### Identification of differentially methylated regions (DMRs)

Differentially methylated regions (DMRs) comprised multiple consecutive methylated CpG sites deemed under similar regulatory control, which has been suggested to be more informative for diseases etiologies. We conducted DMR analysis with the CpG sites associated with smoking history at raw *p*-value < 0.001 with the R package ‘*dmrff*’^24^. We specified the maximum distance to 1500 bp for features considered to be proximal consecutive CpG sites for candidate DMRs. To identify the regional differential methylation among candidate regions, *dmrff* uses an extension of inverse-variance weighted meta-analysis to produce a weighted average difference in DNAm for each candidate DMR. Regional test-statistics were performed and those DMRs with FDR < 0.05 were considered to be statistically significant.

#### Identification of over-representation of genes in biological pathways and gene ontology (GO) terms

To gain more context of the biological activity of former smoking-associated CpG sites, we performed over-representation analyses to identify enrichment pathways and GO terms among the genes annotated to CpG sites with suggestive association at raw *p*-value < 10^–4^ from EWAS. R package ‘*org.Hs.eg.db*’ was used to convert genes to *Entrez* IDs. We used R packages ‘*clusterProfiler*’, ‘*ReactomePA*’, and ‘*DOSE*’ for KEGG pathway^25^ and GO term analysis, REACTOME pathway analysis, and disease ontology semantic and enrichment analysis, respectively. Results within a 10% BH-corrected FDR were considered significant over-representation.

#### Examination of correlation between the time since quitting smoking and DNAm at the significant CpG sites among former smokers

We also examined the association between DNAm at individual genome-wide significant CpG sites and time since smoking cessation among the former smokers. We regressed methylation levels at each significant CpG on time since quitting smoking (years) and three surrogate variables. We then used “*visreg*” package to visualize the partial correlation between each CpG and time since quitting smoking, conditioning on three surrogate variables.

### Ethics approval and consent to participate

The institutional review boards of the Massachusetts General Hospital and Harvard T.H. Chan School of Public Health approved this study. All subjects met with trained study staff before providing written informed consent.

## Results

### Characteristics of the study population

A total of 132 women participating in the EARTH study had a preserved FF sample available for analysis. One sample was damaged during transit. DNA extraction yielded from the remaining 131 samples exhibited a range from 0 to 2896 ng. Only 60 women had DNA extractions of ≥ 200 ng, while 16 had extractions ranging from 100 to 199 ng. We included 64 samples on the DNAm array, including four with DNA extractions yielded between 100 and 199 ng. We excluded two samples that had more than 5% of probes yielding detection *p*-values > 0.001 and another sample that was an outlier in principal components analyses. Furthermore, we excluded one participant who was a current smoker at enrollment into EARTH and one participant with missing covariate data. Consequently, the analytic dataset comprised 474,545 probes from 59 samples.

The 59 women in our study had a mean (range) age of 35 (28–44) years at enrollment, of which 86% were white. The mean BMI was 25 kg/m^2^. 75% were never smokers and 25% were former smokers. The time since smoking cessation among the 15 former smokers ranged from 1 to 17 years. 73%, 17%, and 10% of participants received luteal phase agonist, flare, and antagonist protocols, respectively. The overall education level was high, with 37% of women having completed a college degree and 56% having completed a graduate degree (Table 1).

**Table 1:** Characteristics of fifty-nine women undergoing assisted reproductive technology.

Variable	Never	Former	Overall
	(n = 44)	(n = 15)	(n = 59)
Age (years), mean (SD)	35.5 (4.3)	35.0 (3.5)	35.3 (4.1)
BMI (kg/m^2^), mean (SD)	25.2 (5.4)	24.7 (3.0)	25.1 (4.9)
Time since smoking cessation (years), mean (SD)	NA	9.7 (4.8)	NA
Average daily cigarette consumption throughout the smoking period	NA	5.50 (5.49)	NA
Ovarian stimulation protocol, n (%)			
Antagonist	5 (11.4)	1 (6.7)	6 (10.2)
Flare	7 (15.9)	3 (20.0)	10 (16.9)
Luteal phase agonist	32 (72.7)	11 (73.3)	43 (72.9)
Race, n (%)			
White	38 (86.4)	13 (86.7)	51 (86.4)
Asian	5 (11.4)	0 (0)	5 (8.5)
Other	1 (2.3)	2 (13.3)	3 (5.1)
Education level, n (%)			
Did not graduate from college	0 (0)	4 (26.7)	4 (6.8)
College graduate	16 (36.4)	6 (40.0)	22 (37.3)
Graduate degree	28 (63.6)	5 (33.3)	33 (55.9)

*SD* standard deviation, *BMI* body mass index.

### Identification of former smoking-associated CpGs

**Figure 1:**
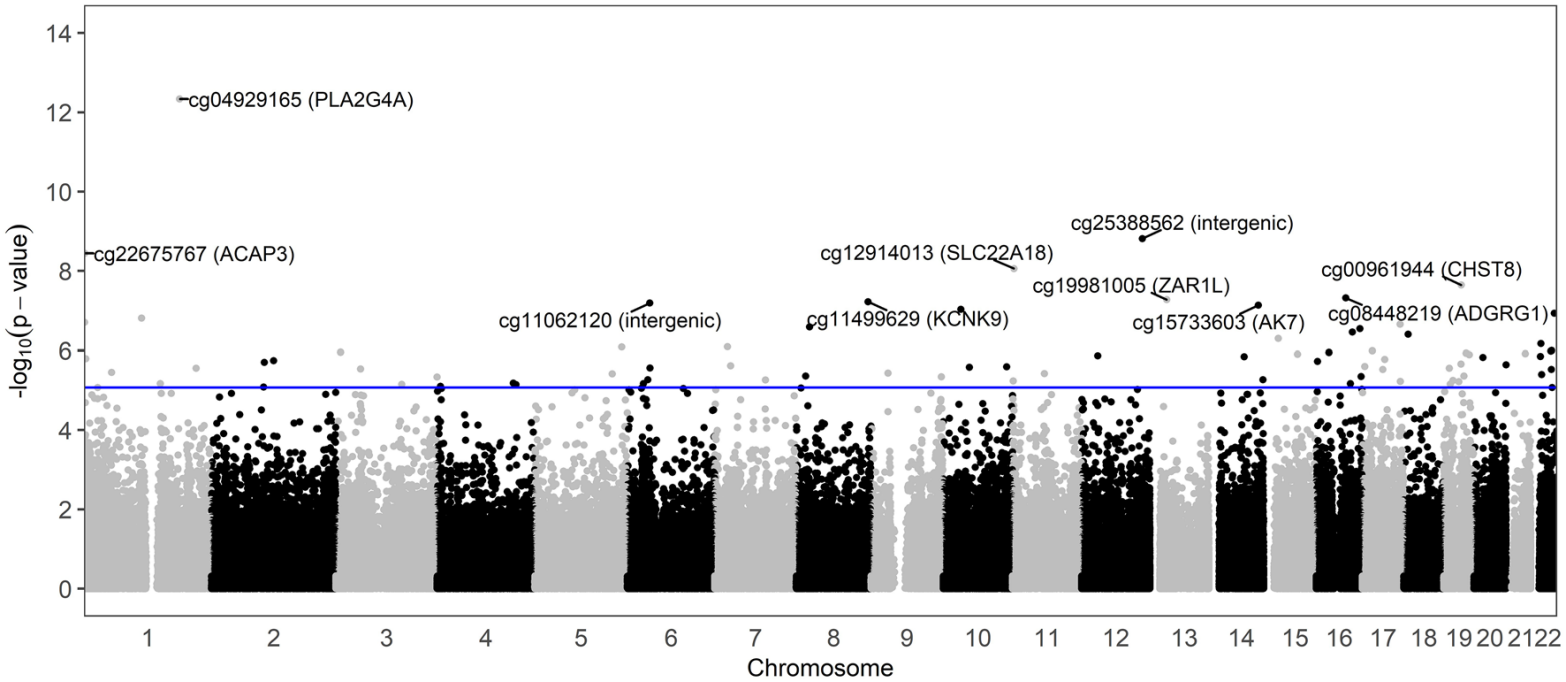
A Manhattan plot of the epigenome-wide association study (EWAS) results. All models were adjusted for age at enrollment, ovarian stimulation protocol, and three surrogate variables. The blue line represents the false discovery rate (FDR) of 5%. The annotated dots include the top 10 most statistically significant former smoking-associated CpGs (gene).

We identified 81 CpGs that were differentially methylated with former smoking at a 5% FDR (Excel Table S1), which were spread throughout the genome (Fig. 1). Comparing former smokers to never smokers, 63 CpGs had higher methylation levels with the effect sizes ranging between 1.0 and 8.0%, while 18 had lower methylation with the effect sizes ranging from − 2.1 to − 7.1% in former smokers. Except for 12 CpGs that were intergenic, the remaining 69 CpGs were within different genes. We also explored whether adjustment for additional potential confounders may explain our findings. Further adjustment for education levels and BMI did not have large impacts on effect size of the 81 significant probes (Fig. S1). Among the top 10 most statistically significant associations from EWAS, two CpGs were intergenic while the other eight CpGs were within *PLA2G4A*, *ACAP3*, *SLC22A18*, *CHST8*, *ADGRG1*, *ZAR1L*, *KCNK9*, and *AK7* (Table 2). The full EWAS results are provided (Excel Table S2).

**Table 2:** Top 10 most statistically significant former smoking-associated CpGs.

CpG ID	Effect size (%)	Std. err	*p*-value	FDR	Chr	Pos	Gene	Region
cg04929165	1.6	0.0023	4.62e−13	< 0.0001	chr1	186807235	*PLA2G4A*	5'UTR
cg25388562	6.6	0.0109	1.53e−09	0.0004	chr12	119060216	–	–
cg22675767	3.5	0.0059	3.57e−09	0.0006	chr1	1234367	*ACAP3*	Body
cg12914013	4.7	0.0082	8.7e−09	0.0010	chr11	2938034	*SLC22A18*	Body
cg00961944	4.8	0.0085	2.25e−08	0.0021	chr19	34245882	*CHST8*	Body
cg11062120	2.8	0.0052	6.48e−08	0.0034	chr6	43851620	–	–
cg11499629	6.1	0.0113	5.96e−08	0.0034	chr8	140646360	*KCNK9*	Body
cg19981005	− 3.0	0.0056	5.28e−08	0.0034	chr13	32877922	*ZAR1L*	3'UTR
cg08448219	1.9	0.0035	4.81e−08	0.0034	chr16	57695718	*ADGRG1*	Body
cg15733603	− 6.2	0.0115	7.29e−08	0.0035	chr14	96863448	*AK7*	Body

The model was adjusted for age at enrollment, ovarian stimulation protocol, and three surrogate variables.

*CpG* cytosin-phosphate-guanine site, *Std. err* standard error, *FDR* false discovery rate adjusted *p*-value, *Chr* chromosome, *Pos* genomic position.

### Identification of DMRs

**Figure 2:**
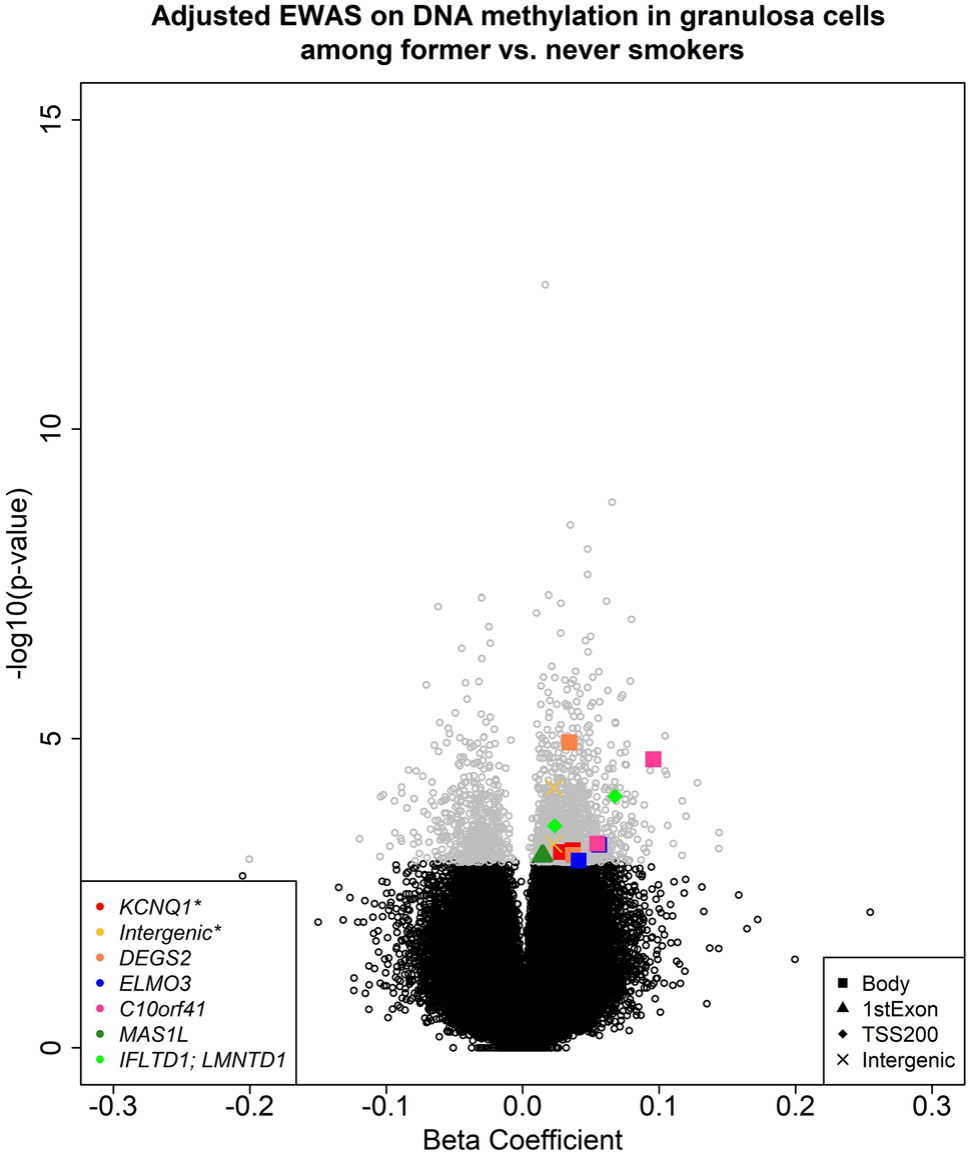
A volcano plot of the beta coefficients and -log_10_ (*p*-values) from the epigenome-wide association study (EWAS). All models were adjusted for age at enrollment, ovarian stimulation protocol, and three surrogate variables. Grey dots denote CpGs that were associated with former smoking at raw *p*-value < 0.001 from EWAS. Different colors highlight significant CpGs within two significant differentially methylated regions (DMRs) with FDR < 0.05 and five additional DMRs with raw *p*-value < 10^–5^. Different shapes represent the regulatory annotations for the CpGs that are highlighted from the DMR analysis and * denotes the DMRs with FDR < 0.05.

We identified 16 candidate DMR regions with at least two differentially methylated CpGs, two of which were significant DMRs with FDR < 0.05 and additional five of which were with raw *p*-value < 10^–5^. The most significant DMR (FDR = 4.01 × 10^–11^) was within *KCNQ1* gene. The other significant DMR (FDR = 1.01 × 10^–6^) was intergenic, but was 4 kb upstream of *RHBDD2*. The additional five DMRs with raw *p*-value < 10^–5^ were within genes *DEGS2*, *ELMO3*, *C10orf41*, *MAS1L*, and *LMNTD1* (Table 3). Significant smoking-associated CpGs within these identified DMRs were all hypermethylated among former versus never smokers (Fig. 2, Excel Table S3).

**Table 3:** Two significant differentially methylated regions (DMRs) with FDR < 0.05 and five additional DMRs with raw *p*-value < 10^–5^.

DMR	Number of CpGs	Coef	Std.Err	*p*-value	FDR	Gene	Region
chr11:2746420–2747204	3	0.1337	0.0161	8.43e−17	4.01e−11	*KCNQ1*	Body
chr7:75522467–75523398	2	0.0978	0.0139	2.12e−12	1.01e−06	*RHBDD2*^a^	
chr14:100621848–100622050	2	0.1291	0.0274	2.46e−06	1e+00	*DEGS2*	Body
chr16:67233748–67233861	3	0.2486	0.0530	2.72e−06	1e+00	*ELMO3*	Body
chr10:77165618–77165750	3	0.4724	0.1019	3.58e−06	1e+00	*C10orf41*	Body
chr6:29454888–29455532	9	0.2612	0.0575	5.52e−06	1e+00	*MAS1L*	1stExon
chr12:25801522–25801556	2	0.2011	0.0449	7.49e−06	1e+00	*IFLTD1;* *LMNTD1*	TSS200

*Chr* chromosome, *CpG* cytosin-phosphate-guanine site, *Coef.* Coefficients, *Std. err* standard error, *FDR* false discovery rate adjusted *p*-value.

^a^The DMR was intergenic. *RHBDD2* was a nearby gene at the upstream of the DMR with the distance of 4 kb.

### Identification of biological pathways and GO terms over-represented in gene set

For the pathway enrichment analyses, we used a list of genes annotated to the CpGs that yielded *p*-value < 0.0001, among which 260 matched to *Entrez* gene IDs. Five KEGG pathways and 24 GO terms were significantly over-represented (FDR < 0.1). The five KEGG pathways are oxytocin signaling pathway (hsa: 04921), adrenergic signaling in cardiomyocytes (hsa: 04261), platelet activation (hsa: 04611), axon guidance (hsa: 04360), and chemokine signaling pathway (hsa: 04062), which contained 9, 8, 7, 8, and 8 genes from our gene set, respectively (Table 4). 24 GO terms were related to general biological processes and molecular functions, which were mainly channel activity, membrane transporter and antiporter activity, oxidoreductase activity, and G protein activity (Fig. S2, Excel Table S4).

**Table 4:** KEGG pathways (FDR < 0.1) enriched with genes annotated to 376 CpGs that were associated with former smoking at *p*-value < 0.0001 from the epigenome-wide association study.

Pathway ID	KEGG pathway description	FDR	Gene^a^
hsa04921	Oxytocin signaling pathway	0.0209	*CACNA1D, RYR1, MYLK3, CAMK2B, PIK3R5,* *PLA2G4A, MYLK, RHOA, HRAS*
hsa04261	Adrenergic signaling in cardiomyocytes	0.0522	*KCNQ1, CREM, CACNA1D, PPP2R2A, PPP2R2C,* *CAMK2B, PIK3R5, PPP2R2D*
hsa04611	Platelet activation	0.0522	*MYLK3, PIK3R5, PLA2G4A, MYLK, RAP1A, RHOA, GP1BA*
hsa04360	Axon guidance	0.0846	*EPHB4, PARD3, PLXNB1, ABLIM2, CAMK2B,* *SRGAP3, RHOA, HRAS*
hsa04062	Chemokine signaling pathway	0.0948	*CX3CL1, JAK3, PARD3, PIK3R5, RAP1A, BCAR1, RHOA, HRAS*

The epigenome-wide association study was adjusted for age at enrollment, ovarian stimulation protocol, and three surrogate variables.

*KEGG* Kyoto encyclopedia of genes and genomes, *FDR* false discovery rate adjusted *p*-value.

^a^The intersection of genes that were present both in the gene list and in within each KEGG pathway.

### Correlation between the time since quitting smoking and DNAm at the significant CpG sites among former smokers

**Figure 3:**
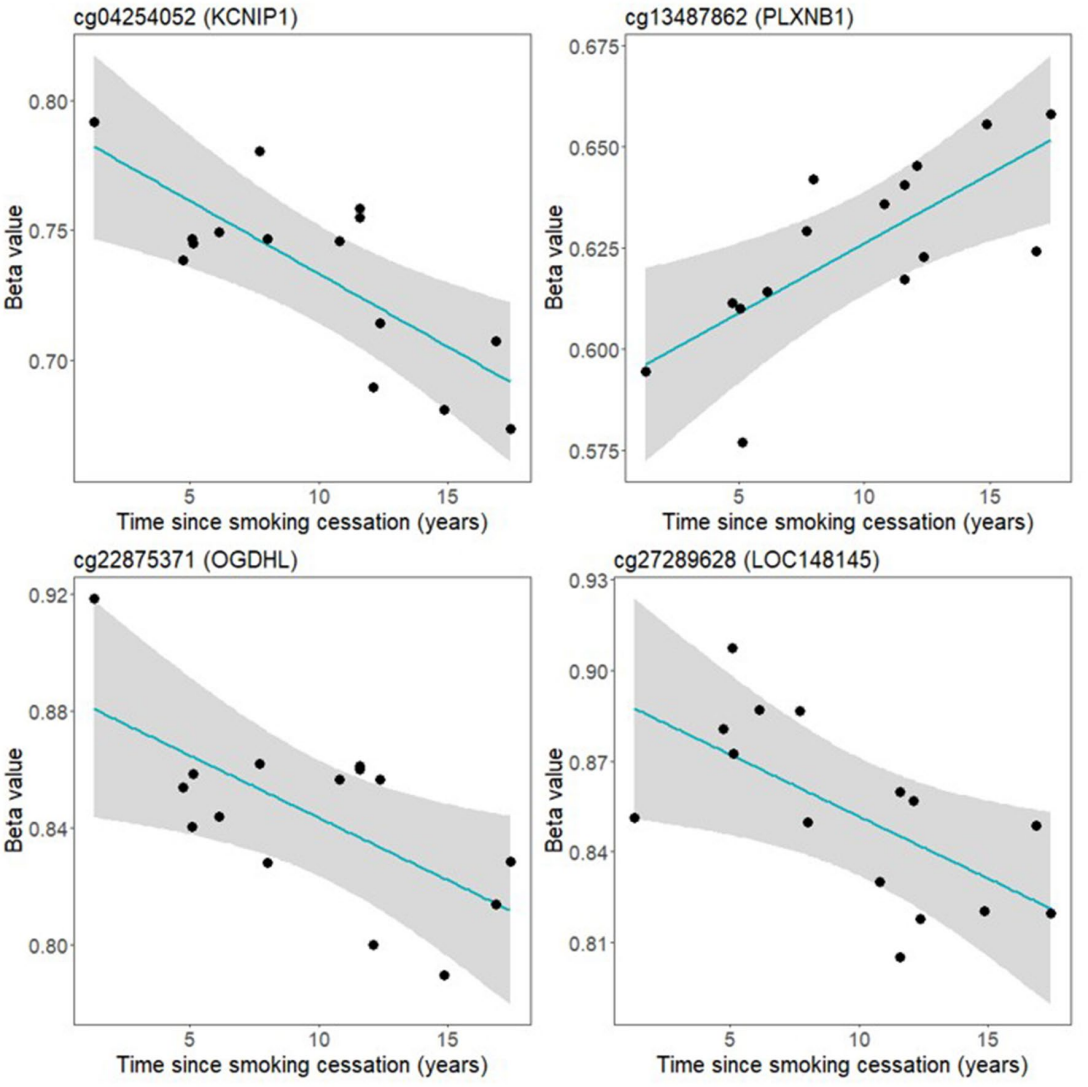
Partial correlations between each of four former smoking-associated CpGs and the time since smoking cessation among 15 former smokers. The model was conditioned on three surrogate variables.

Last, we explored whether the DNAm levels for each of 81 significant CpGs was associated with time since smoking cessation, among the subset of former smokers (n = 15). We regressed the methylation levels at each significant CpG on the time since quitting (years), while controlling for three surrogate variables using linear regression. As a result, four CpGs were significantly associated with the time since smoking cessation while controlling for three surrogate variables (*p* < 0.05), which were cg04254052 (*KCNIP1*) ($\beta$= − 0.006, *p* = 0.004), cg13487862 (*PLXNB1*) ($\beta$ = 0.003, *p* = 0.007), cg22875371 (*OGDHL*) ($\beta$ = − 0.004, *p* = 0.022), cg27289628 (*LOC148145*) ($\beta$ = − 0.004, *p* = 0.025) (Excel Table S5). In other words, a one-year increase in the time since smoking cessation was associated with 0.6%, 0.4%, and 0.4% decrease in the methylation levels at cg04254052 (*KCNIP1*), cg22875371 (*OGDHL*), and cg27289628 (*LOC148145*) respectively, and a 0.3% increase in methylation levels at cg13487862 (*PLXNB1*). For all four of the CpGs where we observed associations with time since quitting (Fig. 3), former smokers had higher DNA methylation levels than never smokers. For three of these four CpGs (annotated to *KCNIP1, OGDHL*, and *LOC148145*), participants that quit smoking more recently also had higher DNA methylation levels than those with increased time since cessation.

## Discussion

We performed an epigenome-wide association study to evaluate the former smoking-associated variations in DNA methylation that occur in GCs among 59 women undergoing ART. We identified 81 CpGs that were differentially methylated among former smokers compared to never smokers. Additionally, we observed evidence of regional differences in DNAm at the *KCNQ1* gene and in one intergenic region upstream of *RHBD2*. Five KEGG pathways were over-represented among the gene set, including oxytocin signaling pathway, adrenergic signaling in cardiomyocytes, platelet activation, axon guidance, and chemokine signaling pathway. Overall, these epigenetic changes have been implicated in the perturbation of biological processes, including those associated with inflammatory responses, reproductive outcomes, cancer development, neurodevelopmental disorders, and cardiometabolic health. To our knowledge, this is the first epigenome-wide association study on prior smoking exposure in human GCs.

Notably, multiple genes annotated to the former smoking associated CpGs that we identified have been reported in association with former and/ or current smoking in a meta-analysis on epigenetic modification of cigarette smoking with 15,907 blood derived samples from participants in 16 cohorts^11^. These genes include *ACAP3*, *KCNK9*, *CHST8*, *PARD3*, *TNRC6B*, *CD37*, and several others. We observed a nominal association between former smoking and DNAm changes on cg05575921 within *AHRR*, a well-documented smoking-associated gene^11,26,27^, with a *p*-value of 0.037 (Excel Table S2), but this did not survive the false discovery rate correction threshold (FDR < 0.05). Additionally, we observed the association between time of quitting smoking and methylation differences at five other CpGs within the *AHRR* gene (*p*-value < 0.05) (results not shown), but none of them were significantly associated with former smoking in EWAS (FDR < 0.05). This may implicate a power issue. The other possibility could be the uniqueness of our tissue sample, as many DNAm associations are tissue-specific^28^. Results should be interpreted with caution. Future large-scale investigations on the influences of smoking on DNAm in GC cells should be performed to validate our findings.

The genes annotated to the top 10 statistically significant CpGs (two were intergenic), were *PLA2G4A*, *ACAP3*, *SLC22A18*, *CHST8*, *KCNK9*, *ZAR1L*, *ADGRG1*, and *AK7*, which may be indicative to potential disruption of several biological processes. *PLA2G4A* is crucial for the composition of eicosanoids including prostaglandins and leukotrienes that play important role in inflammatory responses and hemodynamics regulation^29,30^. *ACAP3* has been observed to be involved in regulating neuronal migration in mice, which suggests its important role in brain development^31^. *SLC22A18* is located in 11p15.5, an important tumor-suppressor gene region, together with other 21 genes including *KCNQ1* that was found within one identified significant DMR. *SLC22A18* encodes a membrane protein, which has been suggested as a tumor suppressor gene in multiple cancers^32–34^, but its function has not been well established. *CHST8* (*GalNAc-4-ST1*) is primarily expressed in the pituitary gland, which is required for the biosynthesis of glycoprotein hormone such as lutropin (LH) and thyrotropin (TSH) by catalyzing sulfate addition of the carbohydrate structures^35^. *KCNK9* encodes an oncogenic potassium (K^+^) channel, which was found overexpressed in both breast, lung, and colorectal cancers^36,37^. It has been suggested to promote tumor growth and metastasis in cancer cell lines^38^. *ZAR1L* (*ZAR2)* was recognized as a maternal factor with function of maintaining the stability of maternal transcriptome^39^, thereby is important for oogenesis and embryogenesis^40^. Besides, *ZAR1L* has been suggested potential associated with tumor suppressors, which can repress the transcription of *BACA2* to repress breast cancer cells^41^. *ADGRG1* (*GPR56*) is a representative tumor-related G protein-coupled receptors (GPCRs) member, which plays an important role in tumor cell growth, migration, angiogenesis, and metastasis^42,43^. *AK7* is known to maintain ciliary structure and function whose dysfunction may lead to primary ciliary dyskinesia including bronchiectasis and reduced fertility^44–46^. In summary, the smoking-associated epigenetic modifications have been associated with tumor development, inflammation, reproductive health, and neurodevelopmental disorders.

We further examined whether the amount of time since smoking cessation correlated with DNAm at the identified CpG sites. Four CpGs showed significant correlations with time since cessation, which were cg04254052 (*KCNIP1*), cg13487862 (*PLXNB1*), cg22875371 (*OGDHL*), and cg27289628 (*LOC148145*). *KCNIP1* is an important component of potassium channel complexes, which regulates neuronal membrane excitability^47,48^. *PLXNB1* is involved in extensive biological processes including cell adhesion, cell migration, and cell shape regulation^49–52^. *OGDHL* is a component of the oxoglutarate dehydrogenase complex that is involved in degradation of glucose and glutamate, and has been associated with tumor development and progression^53,54^. The functions of *LOC148145* are not well characterized at this time. For three of these CpGs (annotated to *KCNIP1*, *OGDHL*, and *LOC148145*), the direction of this association implies that methylation levels may revert back to levels that are consistent with never smokers with increasing time since smoking cessation, which aligns with the findings from studies of other tissue samples. Consistently, previous studies have also reported a gradual reversal of methylation levels at certain smoking-relate CpG sites after quitting smoking, while other CpGs exhibit a more persistent smoking-associated modification^55–57^. Several other CpGs showed potential associations (*p* < 0.1) in our analysis, but these were not statistically significant. We expected more CpGs with reversible methylation after quitting smoking could be identified in a larger sample of former smokers.

We identified two significant smoking-associated DMRs. One DMR (chr11: 2,746,420–2,747,204) contained *KCNQ1* gene. *KCNQ1* contributes significantly to repolarization process, which has been closely linked to cardiometabolic outcomes including cardiac arrhythmias and type 2 diabetes^58,59^. The other DMR (chr7: 75,522,467–75,523,398) is at an intergenic region, upstream of *RHBDD2*, which was found to be overexpressed in breast cancers^60^. In addition, over-representation analysis identified five enriched pathways, including oxytocin signaling pathway, adrenergic signaling in cardiomyocytes, platelet activation, axon guidance, and chemokine signaling pathway. The oxytocin signaling pathway plays a critical role in various aspects of reproductive health^61–64^. Specifically, an increase in synthesis and release oxytocin contributes to the maternal behavior and lactation during pregnancy, and expedites fetal expulsion and reduces postpartum hemorrhage in parturition. Additionally, intermittent oxytocin secretion regulates milk-ejection reflex and initiates maternal behavior in lactation, while disorders in oxytocin secretion have been associated with maternal depression^61,62^. Platelet activation and its controlled activation are crucial for processes including folliculogenesis, ovulation, placental development, implantation, and embryo devlopment^65^. The chemokine signaling pathway is a key process in inflammation. Previous studies have associated several chemokines in FF with ovarian function and successful pregnancy following IVF treatment^66,67^. The smoking-associated CpGs and their corresponding genes identified within these pathways warrants further investigation, which has potential to be developed as screening biomarkers and therapeutic targets for smoking-induced reproductive outcomes. In addition, adrenergic signaling in cardiomyocytes and platelet activation have been associated with adverse cardiovascular outcomes^68–70^, while axon guidance is associated with neuroinflammation^71^. In summary, these pathways were informative to inflammatory responses, reproductive health, neurodevelopmental, and cardiovascular outcomes.

Overall, our analysis identified former smoking-associated CpGs, DMRs, and gene sets that are involved in several biological processes, including inflammation, reproduction, neurodevelopment, cancer, and cardiovascular health. Whether, or how, such epigenetic modifications in GCs may relate to the health outcomes is not yet clear. But our findings align with other published literature regarding smoking-associated DNAm changes in other biosamples including peripheral blood and placenta^6,11^, demonstrating that tobacco smoke has a substantial impact on the epigenome, some of which persists even after smoking cessation, and our study adds that such a signature is detectable in GCs which may have important implications for reproductive health.

The above findings should be interpreted in the contexts of the strengths and limitations of this study. First, GCs are a promising biosample for investigating the associations between environmental exposures and female fertility, because these cells are critical mediators of oocyte health and development. Second, current insurance mandates in Massachusetts, which require women to stop smoking prior to initiating ART, meant that we did not have any current smokers in our study. Nevertheless, our pilot study provided a unique opportunity to study the short- and long-term impacts of smoking cessation on DNAm patterns in GCs. This also allowed us to establish temporality between smoking and the observed variations in DNAm. However, there are some limitations of this analysis. First, we were not able to perform cell sorting or to adjust for cellular composition given that there is no epigenome reference for FF. We also did not adjust for some potential confounders such as alcohol or other substance usage. Rates of alcohol and substance use were too low in our sample to have impacted our findings, but are likely considerations for future studies with larger sample sizes. However, we performed surrogate variable analysis, to capture and adjust for the potential impacts of cellular composition and other unmeasured confounders, which is a common approach for EWAS performed on tissues where reference methylomes are not available. Second, this pilot study has a small sample size which limited statistical power, and this is particularly apparent for our exploratory correlation analyses of time since cessation. Therefore, the interpretation and extrapolation of the results should be cautious. Further investigation on reversibility or persistence of smoking-induced epigenetic changes is necessary. Additionally, due to the uniqueness of our samples, we could not identify an appropriate data source for external validation, but we did use bootstrapping to examine the internal validity of our most significant findings, and found that our results were robust and consistent within our small sample. Future studies could incorporate additional measures of biological activity, such as gene or protein expression, to better delineate the affected underlying biological process, which currently remain unclear. Findings from our analysis should also be replicated or expanded upon in studies with larger sample sizes. It will also be of interest to include current smokers in future studies.

## Conclusions

In conclusion, our study provides evidence regarding the associations between former smoking with DNAm in GCs. The observed smoking-associated epigenetic modifications in GCs have been associated with similar traits as those reported in other studies with different biosamples, including inflammatory responses, reproductive outcomes, cancer development, neurodevelopmental disorders, and cardiometabolic health. Findings are informative to the potential biological pathways that may underlie the documented association between smoking and female infertility. It is also possible our results have implications for biomarker discovery for smoking-associated reproductive health. Though it is unclear whether the DNAm differences that we detected in GCs have direct influences on pregnancy outcomes or offspring health, future investigations are encouraged.

### Supplementary Information

Supplementary Figures.

Supplementary Tables.

## Data Availability

The data that support the findings of this study are available on request from the corresponding author. The data are not publicly available due to privacy or ethical restrictions.
