# Supplementary material for: Former smoking associated with epigenetic modifications in human granulosa cells among women undergoing assisted reproduction
Source: Sci Rep. 2024 Feb 29;14:5009. doi: 10.1038/s41598-024-54957-2 (PMC10904848; doi:10.1038/s41598-024-54957-2)
Supplement: Supplementary file 1 — Supplementary Figures. [file 41598_2024_54957_MOESM1_ESM.docx]

**Supplemental Materials**

**Former smoking associated with epigenetic modifications in human granulosa cells among women undergoing assisted reproduction**

Ziyin Tang,^1^ Audrey J. Gaskins,^2^ Robert B. Hood,^2^ Jennifer B. Ford,^3^ Russ Hauser,^3^ Alicia K. Smith,^4^ Todd M. Everson^1*^

^1^Gangarosa Department of Environmental Health, Rollins School of Public Health, Emory University, Atlanta, GA, United States

^2^Department of Epidemiology, Rollins School of Public Health, Emory University, Atlanta, GA, United States

^3^Department of Environmental Health, Harvard T.H. Chan School of Public Health, Harvad University, Boston, MA, United States

^4^Department of Obstetrics and Gynecology, School of Medicine, Emory University, Atlanta, GA, United States

*Corresponding author:

Todd M. Everson, PhD

Assistant Professor, Gangarosa Department of Environmental Health

Rollins School of Public Health, Emory University

1518 Clifton Rd NE

Atlanta, GA, 30322

<todd.m.everson@emory.edu>

**Figure S1.** Correlation between the coefficients for significant CpGs (*N* = 81) from main analysis and coefficients with further adjustment of body mass index and education level.

The main epigenome-wide analyses were adjusted for age at enrollment, ovarian stimulation protocol, and three surrogate variables.

**Figure S2.** Gene Ontology (GO) terms (FDR < 0.1) significantly associated with genes annotated to 376 CpGs that were associated with former smoking at raw p-value < 0.0001 from epigenome-wide association study.

The epigenome-wide association study was adjusted for age at enrollment, ovarian stimulation protocol, and three surrogate variables.

Note: BP, biological process; MF, molecular function; GeneRatio, the number of genes in the input gene set that were identified in the GO term divided by the total number of genes in that GO term.

**Excel Table S1.** Eighty-one differentially methylated CpGs identified in former smokers compared to never smoker (FDR < 0.05).

The epigenome-wide association study models were adjusted for age at enrollment, ovarian stimulation protocol, and three surrogate variables.

**Excel Table S2.** The full results of epigenome-wide association study of former smoking.

The models were adjusted for age at enrollment, ovarian stimulation protocol, and three surrogate variables.

**Excel Table S3.** CpGs within two significant differentially methylated regions (DMRs) with FDR < 0.05 and five additional DMRs with raw p-value < 10^-5^.

Note: Not all of listed CpGs within DMRs was significantly associated with former smoking from epigenome-wide association study.

**Excel Table S4.** Gene Ontology (GO) terms (FDR < 0.1) significantly associated with genes annotated to 376 CpGs that were associated with former smoking at raw p-value < 0.0001 from epigenome-wide association study.

The epigenome-wide association study was adjusted for age at enrollment, ovarian stimulation protocol, and three surrogate variables. *^a^*The intersection of genes that were present both in the gene list and in within each GO term. Note: FDR, false discovery rate.

**Excel Table S5.** Associations between significant CpGs from epigenome-wide association study (FDR < 0.5) and the time since smoking cessation among 15 former smokers.

We regressed methylation levels at each significant CpG on time since quitting smoking (years) and three surrogate variables. Note: CI, confidence interval; SD, standard deviation.


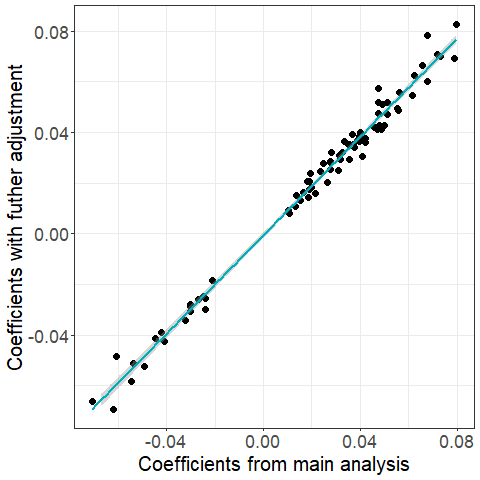


**Figure S1.** Correlation between the coefficients for significant CpGs (*N* = 81) from main analysis and coefficients with further adjustment of body mass index and education level. The main epigenome-wide analyses were adjusted for age at enrollment, ovarian stimulation protocol, and three surrogate variables.

**
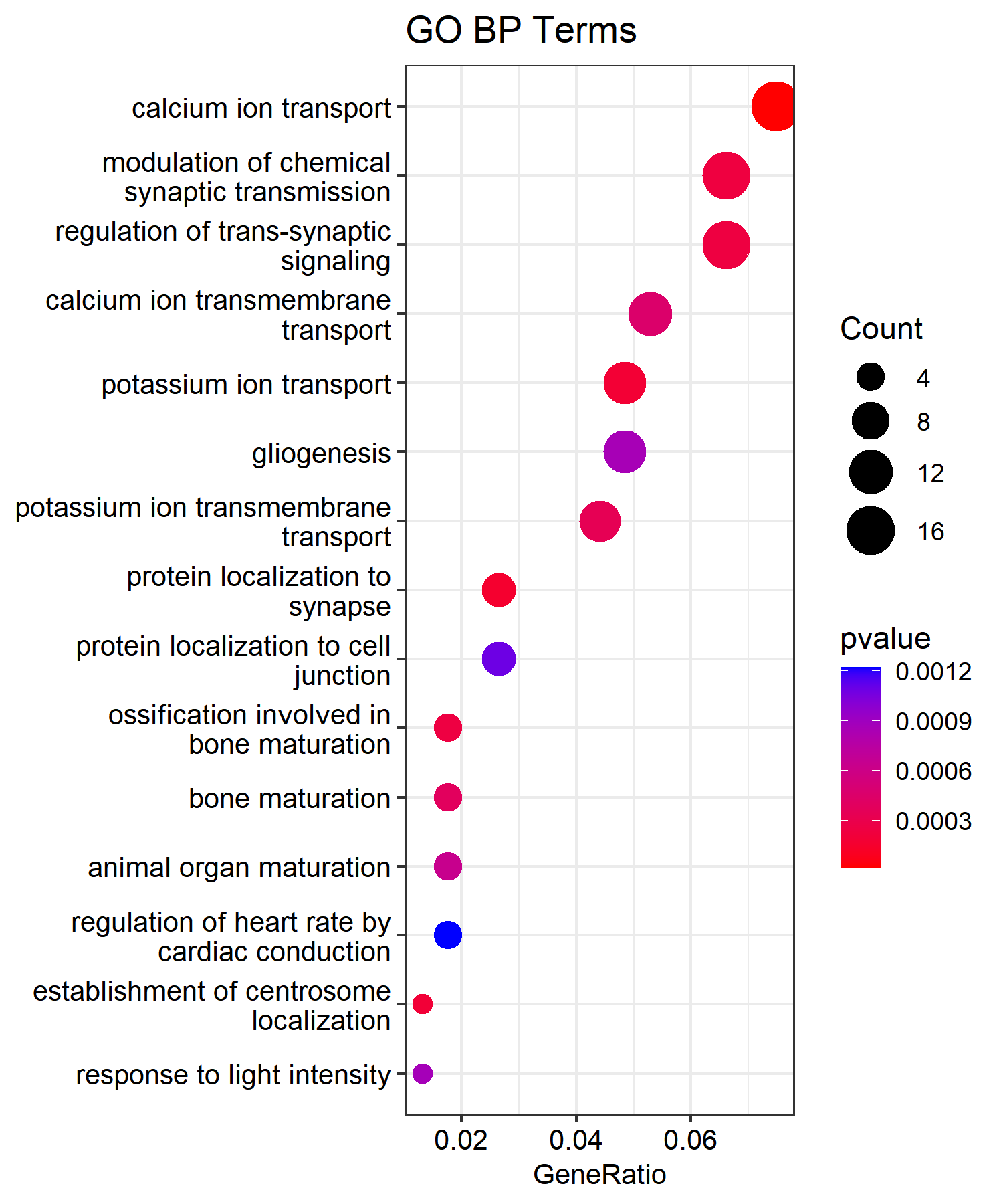

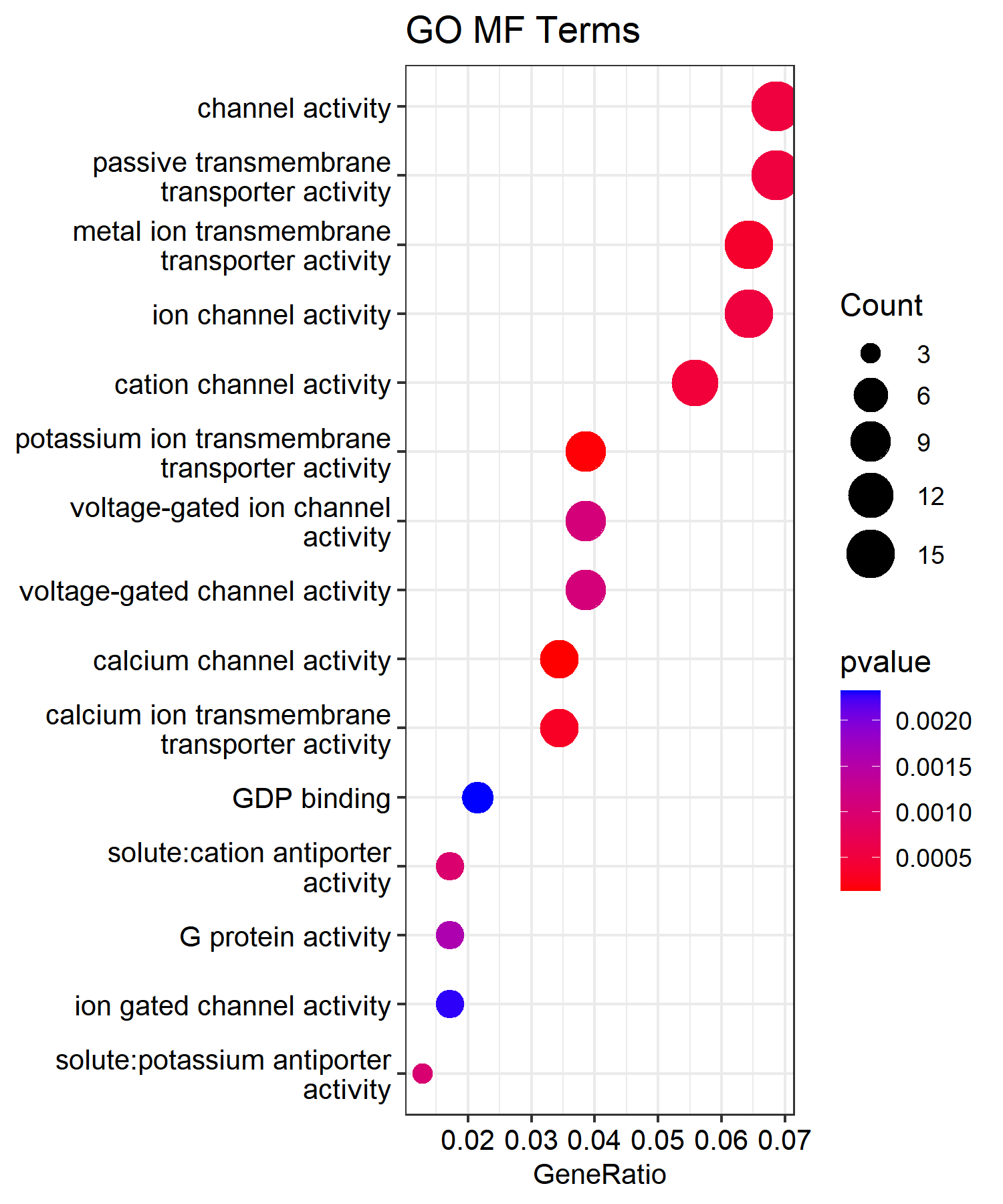
**

**Figure S2.** Gene Ontology (GO) terms (FDR < 0.1) significantly associated with genes annotated to 376 CpGs that were associated with former smoking at raw p-value < 0.0001 from epigenome-wide association study. The epigenome-wide association study was adjusted for age at enrollment, ovarian stimulation protocol, and three surrogate variables.

Note: BP, biological process; MF, molecular function; GeneRatio, the number of genes in the input gene set that were identified in the GO term divided by the total number of genes in that GO term.
